# Chemical Composition and Antioxidant Activity of Essential Oils from Leaves of Two Specimens of *Eugenia florida* DC.

**DOI:** 10.3390/molecules26195848

**Published:** 2021-09-27

**Authors:** Oberdan Oliveira Ferreira, Celeste de Jesus Pereira Franco, Everton Luiz Pompeu Varela, Sebastião Gomes Silva, Márcia Moraes Cascaes, Sandro Percário, Mozaniel Santana de Oliveira, Eloisa Helena de Aguiar Andrade

**Affiliations:** 1 Programa de Pós-Graduação em Biodiversidade e Biotecnologia-Rede Bionorte, Instituto de Ciências Biológicas, Universidade Federal do Pará, Rua Augusto Corrêa S/N, Guamá, Belém 66075-900, Pará, Brazil; oberdan@museu-goeldi.br (O.O.F.); evertonlpvarela@gmail.com (E.L.P.V.); percario@ufpa.br (S.P.); eloisa@museu-goeldi.br (E.H.d.A.A.); 2 Laboratório Adolpho Ducke, Coordenação de Botânica, Museu Paraense Emílio Goeldi, Av. Perimetral, 1901, Terra Firme, Belém 66077-830, Pará, Brazil; celeste.frango12@gmail.com; 3 Laboratório de Pesquisas em Estresse Oxidativo, Instituto de Ciências Biológicas, Universidade Federal do Pará, Rua Augusto Corrêa S/N, Guamá, Belém 66075-900, Pará, Brazil; 4 SEDUC—Secretaria de Estado de Educação, Av. Augusto Montenegro, S/N, Km 10, Icoaraci, Belém 66820-000, Pará, Brazil; professebastiao@yahoo.com.br; 5 Programa de Pós-Graduação em Química, Universidade Federal do Pará, Rua Augusto Corrêa S/N, Guamá, Belém 66075-900, Pará, Brazil; cascaesmm@gmail.com

**Keywords:** natural products, volatile compounds, free radicals, inhibition potential

## Abstract

*Eugenia florida* DC. belongs to the *Myrtaceae* family, which is present in almost all of Brazil. This species is popularly known as *pitanga-preta* or *guamirim* and is used in folk medicine to treat gastrointestinal problems. In this study, two specimens of *Eugenia florida* (Efl) were collected in different areas of the same region. Specimen A (EflA) was collected in an area of secondary forest (capoeira), while specimen B (EflB) was collected in a floodplain area. The essential oils (EOs) were extracted from both specimens of *Eugenia florida* by means of hydrodistillation. Gas chromatography coupled to mass spectrometry (GC/MS) was used to identify the volatile compounds present, and the antioxidant capacity of the EOs was determined by antioxidant capacity (AC-DPPH) and the Trolox equivalent antioxidant (TEAC) assay. For *E. florida*, limonene (11.98%), spathulenol (10.94%) and α-pinene (5.21%) were identified as the main compounds of the EO extracted from sample A, while sample B comprised selina-3,11-dien-6α-ol (12.03%), eremoligenol (11.0%) and γ-elemene (10.70%). This difference in chemical composition impacted the antioxidant activity of the EOs between the studied samples, especially in sample B of *E. florida*. This study is the first to report on the antioxidant activity of *Eugenia florida* DC. essential oils.

## 1. Introduction

In the secondary metabolism of plants, there is the production and accumulation of compounds of different chemical natures [1], and these chemical compounds, called secondary metabolites, are important for the plant’s ability to defend itself against pathogens, predators and environmental stress [2]. The interactions that secondary metabolites exert in plants have awakened great interest in studies, mainly due to the antioxidant properties that some plants have [3].

The search for natural antioxidants, particularly originating from plants, has increased significantly, which may be related to the presence of some compounds of the secondary metabolism of plants that can exhibit oxidizing activity, and, in a way, contribute to the combat and inhibition of free radicals [4], which are involved in the physiopathogenesis of numerous diseases and, especially, degenerative diseases [5,6,7]. In fact, this has been an important aspect in the search for natural antioxidants in essential oils, as these molecules help the performance of various biological activities, as well as assist in the prevention and treatment of various pathologies [8,9].

Additionally, recent studies report the great importance that essential oils have within the food industry [10], as synthetic additives used in the market, such as butylated hydroxyanisol (BHA) and butylhydroxytoluene (BHT), can be responsible for the emergence of human health problems, including hypersensitivity, allergies, asthma, hyperactivity, neurological damage and cancer [11]. However, the addition of essential oils in edible products, either directly or in edible packaging and coatings, can be a valid alternative to preventing autoxidation and prolonging the shelf-life of these products [8].

In this context, it is important to mention that Brazil has the greatest plant biodiversity on the planet, and is highly regarded as a source of natural products. However, much of this plant species biodiversity is still unexplored in regard to its pharmacological potential [12]. Within this wealth of plant species, we can mention the *Myrtaceae* family, which is a large family of edible fruits and comprises about 3800 to 5800 species of woody shrubs or trees, distributed in tropical and subtropical areas [13]. The *Eugenia* genus contained within this family has some fruit species with great nutritional value, due to their antioxidant properties. Furthermore, this genus is considered the fourth largest producer of essential oils within the *Myrtaceae* family [14].

The extraction of essential oils has been performed by several techniques that seek to preserve the integrity of the bioactive compounds and their biological properties, as well as obtaining a good yield. These techniques are classified as either traditional: hydrodistillation, steam distillation, solvent extraction and cold pressing, or innovative: ultrasound-assisted extraction (UAE), microwave-assisted extraction (MAE), pressurized extraction (PLE), and supercritical fluid extraction (SFE) [15,16,17]. Innovative techniques improve the process efficiency in terms of extraction, yield, and in the high quality of the essential oils. In contrast, conventional techniques during the extraction process involve high temperatures and extended extraction times, and these factors can affect the quality of the essential oils by causing chemical changes such as hydrolysis, isomerization, and oxidation [18,19,20].

The *Eugenia florida* DC. is a native species that can be found throughout Brazil, except for Rio Grande do Norte and Sergipe, which presents itself in the form of a tree or a shrub [21]. It is popularly known as *pitanga-preta* or *guamirim*, and its leaves are used in folk medicine as hypotensive, hypoglycemic, antipyretic and in the treatment of gastrointestinal problems [22]. As for the chemical composition of the essential oils of this species, there are few reports in the literature. The study by [23], with a specimen of *Eugenia florida* collected in Rio Grande do Sul, considered an essential oil as part of the sesquiterpene class, whose major compounds were bicyclogermacrene (10.9%), germacrene D (10.4%) and β-caryophyllene (8.1%). Among these compounds, it is important to emphasize that germacrene D and β-caryophyllene are reported to have strong antioxidant activity and radical scavenging capacity, respectively [24]. Given the above, and considering that the antioxidant content of each plant depends on the particular characteristics of the place where the specimens were collected, the present work aimed to evaluate the chemical composition and antioxidant activity of two specimens of *Eugenia florida* found in the Municipality of Magalhães Barata, State of Pará, Brazil.

## 2. Results and Discussion

### 2.1. Yields of the Essential Oils

The obtained contents of the essential oils were 0.20% for the EflA specimen and 0.13% for the EflB specimen. Both specimens were collected in September 2019 in the Municipality of Magalhães Barata, Pará, Brazil, but in different ecosystems. The differences in the yields of the essential oils may be associated with particular aspects related to the sample collection sites [25]. The yields found in this study were very close to those found in studies with other species of the genus, such as *E. uniflora* (0.22–1.68%) [26] and (0.8–3.1%) [27].

### 2.2. Chemical Composition of the Essential Oils

The essential oils of the specimens were obtained by the hydrodistillation technique. Ninety-six chemical components were identified (Table 1), with a total of 93.09% in the EflA specimen and 88.04% in the EflB specimen. The EflA specimen had hydrocarbon (24.17%) and oxygenated (7.72%) monoterpenes, hydrocarbon (18.79%) and oxygenated (41.64%) sesquiterpenes. The EflB specimen showed hydrocarbon (44.06%) and oxygenated (43.98%) sesquiterpenes.

The essential oil from the EflA specimen had limonene (11.98%) as the major component, which was absent in the EflB specimen. This substance is reported in the literature as having antibacterial and antifungal activity against foodborne pathogens [29,30]. In the food industry, for example, it can be used as an inhibitor of yeast growth during the fermentation process [31], and, in addition, this component also presents anti-inflammatory, antioxidant and anticancer activities [32]. Another major component found in the essential oil of the EflA specimen was the oxygenated sesquiterpene spathulenol (10.94%), which was also observed in the EflB specimen (1.2%). It is important to highlight that this substance has antioxidant, anti-inflammatory and antimicrobial activities [33], and insecticidal [34] and antinociceptive [35] activities. The monoterpene α-pinene (5.21%) is present in the EflA oil, but this substance was not identified in the EflB specimen. This monoterpene exists in nature and has (−)-α-pinene and (+)-α-pinene structural enantiomers [36]. In addition, these compounds have demonstrated biological activities, such as being antimicrobial, and are cytotoxic against the cancer cells which cause ovarian cancer [37,38].

The sesquiterpenes caryophyllene oxide (5.0%) and (*E*)-caryophyllene (4.49%) were also identified in the essential oil of the EflA specimen. The (*E*)-caryophyllene, at a lower content (2.35%), was found in the essential oil of the EflB specimen. Studies report that caryophyllene oxide has insecticidal activity against the *Aedes aegypt* vector, an important vector of diseases such as dengue, zika and chikungunya [39]. This compound also presents gastroprotective potential [40] and antiviral potential [41], as well as potential activity against leishmania [42].

The sesquiterpenes selina-3,11-dien-6α-ol (12.03%), eremoligenol (11.0%) and γ-elemene (10.70%) were the main constituents of essential oil EflB. Eremoligenol and γ-elemene were also found in the EflA specimen, but in low concentrations, with contents of 0.71% and 0.16%, respectively. γ-elemene is a sesquiterpene that proves to be toxic to some pest crops, and may be an alternative for the development of new pesticides [43] and insecticides [34,44]. Another compound identified in the essential oils of the specimens was δ-cadinene, with levels of 4.42% for EflB and 3.13% for EflA. It is important to highlight this compound for its acaricide activity [45], antimicrobial activity and as a causative agent of respiratory tract infections, such as pneumonia and sinusitis [46]. The α-cadinol was also found in the essential oils of the specimens, with contents of 4.31% for EflA and of 3.98% for EflB. This oxygenated sesquiterpene has antifungal [47] and cytotoxicity activities against some cancer cell lines [48].

There are few reports of the chemical composition of *E. florida* essential oils in the literature, but this study showed that the chemical composition of the studied specimens differed from each other, in which the specimen (EflB) had a high content of both hydrocarbon and oxygenated sesquiterpenes compared to the specimen (EflA), while the specimen (EflA) showed a high content of monoterpene hydrocarbons. The chemical profile of the studied specimens was different from that found in *E. florida* essential oils by Apel et al. [23]. The variability in the chemical profile of essential oils can be explained by different aspects, such as extraction techniques, climatic and geographical factors, type of soil, light, and temperature [49].

The chemical composition of the studied specimens differed from that found in studies with EOs from species of the genus *Eugenia*, such as, for example, in the study conducted with the *E. uniflora* essential oil, which was characterized by the compounds: selina-1,3,7(11)-trien-8-one (36.37%) and selina-1,3,7(11)-trien-8-one epoxide (27.32%) [50]. The *E. mansoi* essential oil was characterized by (*E*,*Z*)-farnesol (17.3%), (*E*,*E*)-farnesol (14.5%) and viridiflorene (12.5%) [23], and the main compounds for the *E. involucrata* essential oil were elixene (26.53%), β-caryophyllene (13.16%) and α-copaene (8.41%) [51]. The major compounds eugenol (68.9%), (*E*)-caryophyllene (12.6%) and eugenol acetate (12.4%) characterized *E. caryophyllata* EOs [52], and, in addition, the sample of four specimens of *E. biflora* were characterized by the compounds for sample (EBI-1) and (EBI-2): (*E*)-caryophyllene (16.8% and 11.4%) and caryophyllene oxide (28.6% and 20.5%), respectively. The sample (EBI-3) was characterized by cadinol (14.7%) and the sample (EBI-4) by globulol (9.8%), germacrene B (7.9%), and γ-elemene (3.1%) [53].

### 2.3. Antioxidant Activity

To measure the antioxidant activity of *Eugenia florida* essential oils, preformed free radicals DPPH^•^ and ABTS^•+^ were used. Table 2 shows the ability of essential oils, which have been extracted from the dried leaves of *Eugenia florida* EflA and EflB specimens, to scavenge free radicals. According to the results, the TEAC of EflA and EflB specimens were 0.456 mM and 0.652 mM, respectively. When compared to the 1 mM concentration of Trolox, EflA specimen showed 45% inhibition of the ABTS^•+^ radical, and EflB 65%. Both activities were below the standard. Additionally, EflA and EflB specimens presented CA-DPPH^•^ of 1.72 mM and 2.14 mM, respectively. According to these results, the DPPH^•^ radical inhibition capacity of the EflA and the EflB specimen was 72% and 114% higher than the Trolox standard (1 mM), respectively.

Based on these data, we observed that the results of the measurement of antioxidant capacity using the ABTS^•+^ radical scavenging capacity test were different from those obtained with the DPPH^•^ radical. According to other studies, there are differences between the results obtained by DPPH^•^ and ABTS^•+^, resulting from the difference in reaction mechanisms that each of these free radicals presents against the antioxidant molecules presented in the samples [54,55]. In analyses using ABTS^•+^, electron transfer can occur, and different antioxidant compounds provide electrons to reduce the radical cation, and despite the antioxidant compounds’ potential, these compounds have time to fully react, allowing a measurement of the total antioxidant capacity. As for the DPPH^•^ radical, the inhibition is based on the transfer reaction of hydrogen atoms, which can occur between antioxidants and peroxyl radicals. In this method, nitrogen radicals are created instead of peroxyl radicals, which are more stable and less transient, favoring their reaction with antioxidant compounds, which can result in higher levels of antioxidant capacity.

Studies indicate that plants of the *Eugenia* species have several natural antioxidants [56,57]. However, no reports were found in the available literature which could be used as parameters for comparison with oils of the same species. Nonetheless, there are reports of this activity for essential oils obtained from other species of the genus *Eugenia*, such as the essential oil of *E. uniflora*, composed mainly of curzerene (50.6%), selina-1,3,7(11)-trien- 8-one (43.1%) and selina-1,3,7(11)-trien-8-one epoxide (30.4%), which presented potential antioxidant activity both by the TEAC and by the DPPH method [58]. Similarly, the essential oil from the leaves of *E. dysenterica*, composed mostly of (−)-elema-1,3,11(13)-trien-12-ol (24.86%), junenol (6.24%) and δ-cadinene (5.33%), also showed antioxidant activity by the DPPH method, of 5.4 mg/mL [59]. In another study, carried out by [60], the essential oil of *E. caryophyllata*, characterized mostly by eugenol (90.3%), (*E*)–caryophyllene (4.83%) and eugenol acetate (1.87%), showed an elimination percentage of 95.6% of antioxidant activity by the method of DPPH, at a concentration of 10,000 μg/mL.

The essential oils of the fresh leaves, shade-dried leaves, kiln-dried leaves, and flowers of *E. klotzschiana,* were characterized by the major compounds α-copaene (10.6%), β-bisabolene (17.4%), α-(*E*)-bergamotene (29.9%) and germacrene D (13.3%). In this study, by the DPPH method, the essential oils isolated from fresh leaves, kiln-dried leaves, shade-dried leaves and flowers presented antioxidant activity ranging from 29.77 µg/mL, 7.61 µg/mL, 6.48 µg/mL, to 5.70 µg/mL, respectively, while by the TEAC method, the variation was 143.85 µM Trolox/g, 106.27 µM Trolox/g, 104.61 µM Trolox/g and 57.81 µM Trolox/g for essential oils isolated from shade-dried leaves, kiln-dried leaves, fresh flowers and leaves, respectively [61].

In the study by Gomes Vidal Sampaio et al. [62], the essential oil of *E. gracilima*, characterized by the compounds germacrene D (16.10%), γ-muurolene (15.60%) and bicyclogermacrene (8.53%), showed antioxidant activity of 15.67 mg/mL by the DPPH method and 15.16 mg/mL by the TEAC method. Essential oils isolated from leaves and fine branches of species of the genus *Eugenia*, such as *E. egensis*, characterized by the major compounds 5-hydroxy-(*Z*)-calamenene (35.8%), (*E*)-γ-bisabolene (35.0) %) and (2*E*,6*E*)-farnesol (34.5%), showed significant antioxidant activity by the TEAC method, which may be related to the presence of the majority compound 5-hydroxy-(*Z*)-calamenene [63]. In another study, the essential oil of *E. neonitida*, rich in hydrocarbon sesquiterpenes, presented a higher percentage of inhibitors than the essential oil isolated from *E. rotundifolia* leaves, rich in hydrocarbon monoterpenes [64]. However, in the essential oil from the leaves of *E. uniflora*, characterized by the major compounds germacrene B (21.2%), selina-1,3,7-(11)-trien-8-one oxide (19.3%) and (*E*)-caryophyllene (12.6%), the antioxidant activity of 833.3 μg/mL by the DPPH method and 8.1 μg/mL by the TEAC method were observed [24].

It is possible that the antioxidant activity of the *E. florida* essential oils is mainly attributed to its main components; for the EflA specimen, these are: limonene (11.98%), spathulenol (10.94%), α-pinene (5.21%), caryophyllene oxide (5.0%) and (*E*)-caryophyllene (4.49%); while for the EflB specimen, these are: selina-3,11-dien-6α-ol (12.03%), eremoligenol (11.0%) and γ-elemene (10.70%). All these molecules are described as presenting antioxidant activities [33,65]. The chemical composition of the essential oils was strongly evidenced by the presence of sesquiterpene hydrocarbons, thus, we can attribute the antioxidant effect to these sesquiterpene compounds [66], especially to the compound (*E*)-caryophyllene, as it is considered an excellent antioxidant [51].

In addition, it is important to emphasize the synergistic interactions present in the chemical constituents of these essential oils, which may also have contributed to the antioxidant activity presented in each of the chemical profiles [26].

## 3. Materials and Methods

### 3.1. Botanical Material

Aerial parts of two specimens of *Eugenia florida* (Efl) were collected on 21 September 2019, in the morning (9:30 a.m.), in the Amazon summer (dry period), in the coastal region of the State of Pará, in the city of Magalhães Barata, Brazil, whose geographic coordinates are 0°48′7.1″ S 47°33′50.3″ W. Specimen A (EflA) was collected in an area of secondary forest (capoeira), while specimen B (EflB) was collected in a floodplain area, on the banks of the Curral river. The exsiccated specimens were added to the Aromatic Plants of the Amazon, Belém, Pará collection of the João Murça Pires Herbarium (MG) in the Emílio Goeldi Museum and were given the following registrations: MG231870 (EflA) and MG237472 (EflB).

### 3.2. Preparation and Characterization of the Botanical Material

The samples from the *Eugenia florida* leaves were dried in a greenhouse with air circulation at a temperature of 35 °C for 5 days, and then shredded in a knife mill (Tecnal, model TE-631/3, Brazil). Moisture content was analyzed using an infrared moisture detector (ID50; GEHAKA, São Paulo, Brazil), in the temperature range of 60 to 180 °C, with 1 °C increments and bidirectional RS-232C output.

### 3.3. Extraction of Essential Oils

The samples were subjected to hydrodistillation in modified Clevenger-type glass systems for 3 h, coupled to a refrigeration system to maintain the condensation water at around 12 °C. After the extraction, the oils were centrifuged for 5 min at 3000 rpm, dehydrated with anhydrous sodium sulfate and centrifuged again under the same conditions. Oil yield was calculated in mL/100 g. The oils were stored in amber glass ampoules, sealed with flame, and stored in a refrigerator at 5 °C.

### 3.4. Chemical Composition Analysis

The chemical compositions of the EOs of *E*. *florida* (A and B), were analyzed using a Shimadzu QP-2010 plus (Kyoto, Japan) a gas chromatography system equipped with an Rtx-5MS capillary column (30 m × 0.25 mm; 0.25 µm film thickness) (Restek Corporation, Bellefonte, PA, USA) coupled to a mass spectrometer (GC/MS) (Shimadzu, Kyoto, Japan). The program temperature was maintained at 60–240 °C at a rate of 3 °C/min, with an injector temperature of 250 °C, helium as the carrier gas (linear velocity of 32 cm/s, measured at 100 °C) and a splitless injection (1 μL of a 2:1000 hexane solution), using the same operating conditions as described in the literature [67,68]. Except for the carrier hydrogen gas, the components were quantified using gas chromatography (CG) on a Shimadzu QP-2010 system (Kyoto, Japan), equipped with a flame ionization detector (FID) (Kyoto, Japan), under the same operating conditions as before. The retention index for all volatile constituents was calculated using a homologous series of n-alkanes (C_8_–C_40_) Sigma-Aldrich (San Luis, USA), according with Van den Dool and Kratz [69]. The components were identified by comparison (i) of the experimental mass spectra with those compiled in libraries (reference) and (ii) their retention indices to those found in the literature [28,70,71].

### 3.5. Trolox Equivalent Antioxidant Capacity (TEAC)

The antioxidant potential of the studied substances was determined according to their equivalence to the potent antioxidant, Trolox (6-hydroxy-2,5,7,8-tetramethylchromono-2-carboxylic acid; Sigma-Aldrich; 23881-3; São Paulo, Brazil), and a water-soluble synthetic vitamin E analogue.

The Trolox equivalent antioxidant capacity (TEAC) was determined according to the methodology adapted from Miller et al. [72] and modified by Re et al. [73]. ABTS^•+^ (2,2’-Azino-bis(3-ethylbenzothiazoline-6-sulfonic acid); Sigma-Aldrich; A1888; São Paulo, Brazil) was prepared using 7 mM ABTS^•+^ and 140 mM of potassium persulfate (K_2_O_8_S_2_; Sigma Aldrich; 216224; São Paulo, Brazil) incubated at room temperature without light for 16 h. Then, the solution was diluted with phosphate-buffered saline until it reached an absorbance of 0.700 (±0.02) at 734 nm.

To measure the antioxidant capacity, 2.97 mL of the ABTS^•+^ solution was transferred to the cuvette, and the absorbance at 734 nm was determined using a Biospectro SP 22 spectrophotometer (São Paulo, Brazil). Then, 0.03 mL of the sample was added to the cuvette containing the ABTS^•+^ radical and, after 5 min, the second reading was performed. The synthetic antioxidant Trolox (6-hydroxy-2,5,7,8-tetramethylchromono-2-carboxylic acid; Sigma Aldrich; 23881-3; São Paulo, Brazil) was used as a standard solution for the calibration curve (*y* = 0.4162x − 0.0023, where y represents the value of absorbance and x, the value of concentration, expressed as mM; R^2^ = 0.9789). The results were expressed as mM. The values found for the samples were compared to the Trolox standard (1 mM).

### 3.6. Antioxidant Capacity by Inhibition of Radical DPPH^•^ (AC-DPPH^•^)

The test was carried out according to the method proposed by [74]. To measure the antioxidant capacity, initially, the absorbance of DPPH^•^ solution (2,2-diphenyl-1-picrylhydrazyl; Sigma-Aldrich; D9132; São Paulo, Brazil) 0.1 mM diluted in ethanol was determined. Subsequently, 0.6 mL of DPPH^•^ solution, 0.35 mL of distilled water and 0.05 mL of the sample were mixed and placed in a water bath at 37 °C for 30 min. Thereafter, the absorbances were determined in a spectrophotometer Bioespectro SP 22 (São Paulo, Brazil) at 517 nm. The synthetic antioxidant Trolox (6-hydroxy-2,5,7,8-tetramethylchromono-2-carboxylic acid; Sigma-Aldrich; 23881-3; São Paulo, Brazil) was used as a standard solution for the calibration curve (*y* = 0.1271x − 0.0023, where y represents the value of absorbance and x, the value of concentration, expressed as mM; R^2^ = 0.9856). The results were expressed as mM. The values found for the samples were compared to the Trolox standard (1 mM).

### 3.7. Statistical Analysis

Results were expressed as the mean of three replicates ± standard deviation of percent inhibition. The activity of essential oils from *E. florida* leaves was analyzed by Student’s T-Test, considering *p* < 0.05 as significant.

## 4. Conclusions

The essential oils from *Eugenia florida* specimens showed different chemical compositions, which may have been influenced by the type of ecosystem where the samples were obtained, i.e., specimen A presented the hydrocarbon monoterpenes and oxygenated monoterpenes classes, predominantly, while specimen B presented hydrocarbon sesquiterpenes and oxygenated sesquiterpenes as major classes. This difference may have affected the potential antioxidant activity of the samples, as specimen B showed superior antioxidant activity for both analyzed methods (TEAC and DPPH). The essential oils of *Eugenia florida* DC. may be a promising source of antioxidant compounds.

## Figures and Tables

**Table 1:** Chemical composition of essential oils extracted from *Eugenia florida* leaves by hydrodistillation.

RI_L_	RI_C_	Constituents	Efl (A)	Efl (B)
932	934	α-Pinene	5.21	
974	977	β-Pinene	3.93	
988	989	Myrcene	0.44	
1002	1005	α-Phellandrene	0.96	
1022	1024	*ortho*-Cymene	1.14	
1024	1030	Limonene	11.98	
1054	1058	γ-Terpinene	0.06	
1086	1089	Terpinolene	0.45	
1095	1099	Linalool	0.39	
1122	1125	α-Campholenal	0.09	
1135	1136	Nopinone	0.05	
1135	1139	*trans*-Pinocarveol	0.86	
1137	1148	*trans*-Verbenol	0.25	
1160	1162	Pinocarvone	0.37	
1166	1166	ρ-Mentha-1,5-dien-8-ol	0.81	
1174	1177	Terpinen-4-ol	0.27	
1186	1191	α-Terpineol	1.37	
1194	1197	Myrtenol	1.01	
1195	1201	*cis*-Piperitol	0.07	
1204	1209	Verbenone	0.42	
1215	1219	*trans*-Carveol	0.63	
1227	1227	*cis-p*-Mentha-1(7),8-dien-2-ol	0.06	
1226	1230	*cis*-Carveol	0.14	
1239	1239	Cumin aldehyde	0.04	
1239	1244	Carvone	0.69	
1249	1253	Geraniol	0.03	
1254	1255	Linalool acetate	0.11	
1269	1274	Perilla aldehyde	0.05	
1287	1287	*trans*-Linalool oxide acetate(pyranoid)	0.06	
1335	1339	δ-Elemene	0.79	0.55
1345	1351	α-Cubebene	0.16	0.05
1359	1364	Neryl acetate	0.05	
1373	1378	α-Ylangene		0.03
1374	1379	α-Copaene	0.54	0.26
1379	1384	Geranyl acetate	0.67	
1387	1387	β-Bourbonene		0.05
1389	1394	β-Elemene	0.4	1.04
1417	1424	(*E*)-Caryophyllene	4.49	2.35
1432	1431	*trans*-α-Bergamotene		1.78
1430	1432	β-Copaene	0.1	
1434	1435	γ-Elemene	0.16	10.70
1439	1442	Aromadendrene	0.13	0.23
1447	1448	Isogermacrene D		0.11
1451	1453	*trans*-Muurola-3,5-diene	0.14	0.31
1452	1457	α-Humulene	0.82	0.39
1458	1464	*allo*-Aromadendrene		0.08
1464	1464	9-*epi*-(*E*)-Caryophyllene	0.44	
1471	1476	Dauca-5,8-diene	0.21	0.64
1478	1479	γ-Muurolene	0.25	0.69
1484	1482	Germacrene D	1.17	2.21
1489	1489	β-Selinene		0.96
1493	1495	*trans*-Muurola-4(14),5-diene		0.51
1496	1498	Viridiflorene		1.55
1500	1500	Bicyclogermacrene	2.12	
1500	1502	α-Muurolene	0.28	0.47
1511	1510	δ-Amorphene	0.24	0.31
1514	1513	(*Z*)-γ-Bisabolene		3.41
1513	1517	γ-Cadinene	0.13	0.61
1531	1523	γ-Vetivenene		1.59
1522	1527	δ-Cadinene	3.13	4.42
1528	1529	Zonarene		0.65
1533	1535	*trans*-Cadina-1,4-diene	0.2	0.36
1545	1539	Selina-4(15),7(11)-diene		0.99
1544	1547	α-Calacorene	1.43	
1554	1553	β-Vetivenene		4.59
1547	1557	Italicene epoxide	1.3	
1559	1562	Germacrene B		2.17
1564	1566	β-Calacorene	0.16	
1567	1572	Palustrol	0.83	
1577	1589	Spathulenol	10.94	1.2
1582	1592	Caryophyllene oxide	5.0	
1590	1592	Globulol	2.32	
1596	1596	Fokienol	0.48	3.9
1592	1599	Viridiflorol	2.43	1.23
1602	1609	Ledol	1.54	
1608	1610	β-Atlantol		0.96
1608	1615	Humulene epoxide II	1.18	
1618	1620	1,10-di-*epi*-Cubenol	0.49	
1642	1622	Selina-3,11-dien-6α-ol		12.83
1629	1628	Eremoligenol	0.71	11.0
1640	1631	*epi*-α-Murrolol	0.27	
1630	1636	Muurola-4,10(14)-dien-1- β-ol	3.52	1.24
1639	1638	Caryophylla-4(12),8(13)-dien-5α-ol	0.31	
1645	1647	Cubenol		2.5
1652	1648	α-Cadinol	4.31	3.98
1653	1652	*allo*-Aromandendrene epoxide		1.51
1649	1655	*cis*-Guaia-3,9-dien-11-ol	0.39	
1668	1665	14-hydroxy-9-*epi*-(*E*)-Caryophyllene	1.53	
1679	1668	Khusinol	0.25	0.96
1682	1678	5-neo-Cedranol	2.25	0.18
1681	1685	Mustakone	1.22	0.07
1686	1688	Germacra-4(15),5,10(14)-trien-1-α-ol	0.11	0.06
1700	1701	Eudesm-7(11)-en-4-ol		1.91
1745	1736	γ-Costol		0.45
1767	1760	14-oxy-α-Muurolene	0.1	
1803	1805	14-hydroxy-δ-Cadinene	0.04	
		Hydrocarbon monoterpenes	24.17	
		Oxygenated monoterpenes	7.72	
		Hydrocarbon sesquiterpenes	18.79	44.06
		Oxygenated sesquiterpenes	41.64	43.98
		Others	0.77	
		Total	93.09	88.04

RI_C_: calculated from a series of n-alkanes (C8–C40) in a DB-5MS column capillary column, RI_L_: [28]. RI_C_: calculated Retention Index; RI_L_: literature Retention Index.

**Table 2:** Antioxidant capacity of essential oils from leaves of *Eugenia florida* specimens.

Specimen	Collection Site	Antioxidant Capacity	
		TEAC (mM)	DPPH (mM)
EflA	Capoeira	0.456 ± 0.005	1.72 ± 0.07
EflB	Banks of the Curral river	0.652 ± 0.023 *	2.14 ± 0.007 ^#^

Values are expressed as mean and standard deviation (n = 3) of antioxidant capacity. TEAC = Trolox Equivalent Antioxidant Capacity; DPPH-AC^•^ s = Antioxidant Capacity by inhibiting the DPPH^•^ radical. (*) and (^#^) means that in the TEAC antioxidant test, the samples are statistically different.

## Data Availability

The data presented in this study are available on request from the corresponding author.

## Sample Availability

The essential oil of the Museu Paraense Emílio Goeldi is available from the authors.

## References

[1] González Mera I.F., González Falconí D.E., Morera Córdova V. (2019). Secondary metabolites in plants: Main classes, phytochemical analysis and pharmacological activities. Bionatura.

[2] Ramakrishna A., Ravishankar G.A. (2011). Influence of abiotic stress signals on secondary metabolites in plants. Plant Signal. Behav..

[3] Salvador M.J., De Lourenço C.C., Andreazza N.L., Pascoal A.C.R.F., Stefanello M.É.A. (2011). Antioxidant capacity and phenolic content of four myrtaceae plants of the South of Brazil. Nat. Prod. Commun..

[4] Pontes F.C., Abdalla V.C.P., Imatomi M., Fuentes L.F.G., Gualtieri S.C.J. (2019). Antifungal and antioxidant activities of mature leaves of *Myrcia splendens* (Sw.) DC. Brazilian J. Biol..

[5] De Souza R.F., Da Silva J.K.R., Da Silva G.A., Arruda A.C., Da Silva M.N., Arruda M.S.P. (2015). Chemical study and evaluation of the antioxidant potential of sapwood of *Vatairea guianensis* Aubl. Rev. Virtual Quim..

[6] Gan R.Y., Xu X.R., Song F.L., Kuang L., Li H. (2010). Bin Antioxidant activity and total phenolic content of medicinal plants associated with prevention and treatment of cardiovascular and cerebrovascular diseases. J. Med. Plants Res..

[7] Pereira R.J., das Cardoso M.G. (2012). Metabólitos secundários e hipertireodismo. J. Biotechnol. Biodivers..

[8] Amorati R., Foti M.C., Valgimigli L. (2013). Antioxidant Activity of Essential Oils. J. Agric. Food Chem..

[9] da Silva L.A., Raposo J.D.A., Campos L.P.G., da Conceição E.C., de Oliveira R.B., Mourão R.H.V. (2018). Atividade antioxidante do óleo essencial de *Myrcia sylvatica* (G. Mey.) DC. por diferentes métodos de análises antioxidantes (ABTS, DPPH, FRAP, β-caroteno/ácido linoleico). Rev. Fitos.

[10] Farias P.K.S., Lopes Silva J.C.R., de Souza C.N., da Fonseca F.S.A., Brandi I.V., Martins E.R., Azevedo A.M., de Almeida A.C. (2019). Antioxidant activity of essential oils from condiment plants and their effect on lactic cultures and pathogenic bacteria. Cienc. Rural.

[11] Anand S.P., Sati N. (2013). Artificial Preservatives and Their Harmful Effects: Looking Toward Nature for Safer Alternatives. Int. J. Pharm. Sci. Res. IJPSR.

[12] Scur M.C., Pinto F.G.S., Pandini J.A., Costa W.F., Leite C.W., Temponi L.G. (2016). Antimicrobial and antioxidant activity of essential oil and different plant extracts of *Psidium cattleianum* Sabine. Braz. J. Biol..

[13] de Araújo F.F., Neri-Numa I.A., de Paulo Farias D., da Cunha G.R.M.C., Pastore G.M. (2019). Wild Brazilian species of Eugenia genera (Myrtaceae) as an innovation hotspot for food and pharmacological purposes. Food Res. Int..

[14] De Souza A.M., De Oliveira C.F., De Oliveira V.B., Betim F.C.M., Miguel O.G., Miguel M.D. (2018). Traditional Uses, Phytochemistry, and Antimicrobial Activities of Eugenia Species—A Review. Planta Med..

[15] Baldino L., Scognamiglio M., Reverchon E. (2020). Supercritical fluid technologies applied to the extraction of compounds of industrial interest from *Cannabis sativa* L. and to their pharmaceutical formulations: A review. J. Supercrit. Fluids.

[16] de Oliveira M.S., Silva S.G., da Cruz J.N., Ortiz E., da Costa W.A., Bezerra F.W.F., Cunha V.M.B., Cordeiro R.M., de Neto A.M.J.C., de Andrade E.H.A., Inamuddin R.M., Asiri A.M. (2019). Supercritical CO_2_ Application in Essential Oil Extraction. Industrial Applications of Green Solvents—Volume II.

[17] Bezerra F.W.F., de Oliveira M.S., Bezerra P.N., Cunha V.M.B., Silva M.P., da Costa W.A., Pinto R.H.H., Cordeiro R.M., da Cruz J.N., Chaves Neto A.M.J. (2020). Extraction of bioactive compounds. Green Sustainable Process for Chemical and Environmental Engineering and Science.

[18] Aziz Z.A.A., Ahmad A., Setapar S.H.M., Karakucuk A., Azim M.M., Lokhat D., Rafatullah M., Ganash M., Kamal M.A., Ashraf G.M. (2018). Essential Oils: Extraction Techniques, Pharmaceutical And Therapeutic Potential—A Review. Curr. Drug Metab..

[19] Santana de Oliveira M., Pereira da Silva V.M., Cantão Freitas L., Gomes Silva S., Nevez Cruz J., Aguiar Andrade E.H. (2021). Extraction Yield, Chemical Composition, Preliminary Toxicity of Bignonia nocturna (Bignoniaceae) Essential Oil and in Silico Evaluation of the Interaction. Chem. Biodivers..

[20] Bagheri H., Abdul Manap M.Y., Bin, Solati Z. (2014). Antioxidant activity of Piper nigrum L. essential oil extracted by supercritical CO2 extraction and hydro-distillation. Talanta.

[21] Mazine F.F., Valdemarin K.S., Bünger M., Faria J.E.Q., Fernandes T., Giaretta A., Santana K.C., Sobral M., Souza M.A. (2020). Eugenia in Flora do Brasil. http://floradobrasil.jbrj.gov.br/reflora/listaBrasil/FichaPublicaTaxonUC/FichaPublicaTaxonUC.do?id=FB10400.

[22] Santos P.F.P., Gomes L.N.L.F., Mazzei J.L., Fontão A.P.A., Sampaio A.L.F., Siani A.C., Valente L.M.M. (2018). Polyphenol and triterpenoid constituents of eugenia Florida dc. (myrtaceae) leaves and their antioxidant and cytotoxic potential. Quim. Nova.

[23] Apel M.A., Sobral M., Schapoval E.E.S., Henriques A.T., Menut C., Bessiere J.M. (2004). Essential oil composition of eugenia florida and eugenia mansoi. J. Essent. Oil Res..

[24] Victoria F.N., Lenardão E.J., Savegnago L., Perin G., Jacob R.G., Alves D., da Silva W.P., da Motta A.D., Nascente P.D. (2012). Essential oil of the leaves of *Eugenia uniflora* L.: Antioxidant and antimicrobial properties. Food Chem. Toxicol..

[25] de Assis A.L.A., Cipriano R.R., Cuquel F.L., Deschamps C. (2020). Effect of drying method and storage conditions on the essential oil yield and composition in *Eugenia uniflora* L. leaves. Rev. Colomb. Ciencias Hortícolas.

[26] Cipriano R.R., Maia B.H.L.N.S., Deschamps C. (2021). Chemical variability of essential oils of *Eugenia uniflora* l. Genotypes and their antioxidant activity. An. Acad. Bras. Cienc..

[27] da Costa J.S., Barroso A.S., Mourão R.H.V., da Silva J.K.R., Maia J.G.S., Figueiredo P.L.B. (2020). Seasonal and Antioxidant Evaluation of Essential Oil from *Eugenia uniflora* L., Curzerene-Rich, Thermally Produced in Situ. Biomolecules.

[28] Adams R.P., Adams R.P. (2007). Identification of Essential Oil Components by Gas Chromatography/Mass Spectroscopy.

[29] Jing L., Lei Z., Li L., Xie R., Xi W., Guan Y., Sumner L.W., Zhou Z. (2014). Antifungal activity of citrus essential oils. J. Agric. Food Chem..

[30] Hąc-Wydro K., Flasiński M., Romańczuk K. (2017). Essential oils as food eco-preservatives: Model system studies on the effect of temperature on limonene antibacterial activity. Food Chem..

[31] John I., Muthukumar K., Arunagiri A. (2017). A review on the potential of citrus waste for D-Limonene, pectin, and bioethanol production. Int. J. Green Energy.

[32] Vieira A.J., Beserra F.P., Souza M.C., Totti B.M., Rozza A.L. (2018). Limonene: Aroma of innovation in health and disease. Chem. Biol. Interact..

[33] do Nascimento K.F., Moreira F.M.F., Alencar Santos J., Kassuya C.A.L., Croda J.H.R., Cardoso C.A.L., do Vieira M.C., Góis Ruiz A.L.T., Ann Foglio M., de Carvalho J.E. (2018). Antioxidant, anti-inflammatory, antiproliferative and antimycobacterial activities of the essential oil of *Psidium guineense* Sw. and spathulenol. J. Ethnopharmacol..

[34] Benelli G., Pavela R., Drenaggi E., Desneux N., Maggi F. (2020). Phytol, (E)-nerolidol and spathulenol from Stevia rebaudiana leaf essential oil as effective and eco-friendly botanical insecticides against *Metopolophium dirhodum*. Ind. Crop. Prod..

[35] Dos Santos E., Radai J.A.S., do Nascimento K.F., Formagio A.S.N., de Matos Balsalobre N., Ziff E.B., Castelon Konkiewitz E., Kassuya C.A.L. (2020). Contribution of spathulenol to the anti-nociceptive effects of *Psidium guineense*. Nutr. Neurosci..

[36] da Silva A.C.R., Lopes P.M., de Azevedo M.M.B., Costa D.C.M., Alviano C.S., Alviano D.S. (2012). Biological Activities of a-Pinene and β-Pinene Enantiomers. Molecules.

[37] da Rodrigues K.A.F., Amorim L.V., Dias C.N., Moraes D.F.C., Carneiro S.M.P., de Carvalho F.A.A. (2015). *Syzygium cumini* (L.) Skeels essential oil and its major constituent α-pinene exhibit anti-Leishmania activity through immunomodulation in vitro. J. Ethnopharmacol..

[38] Hou J., Zhang Y., Zhu Y., Zhou B., Ren C., Liang S., Guo Y. (2019). A-Pinene Induces Apoptotic Cell Death via Caspase Activation in Human Ovarian Cancer. Med. Sci. Monit..

[39] Nararak J., Sathantriphop S., Kongmee M., Mahiou-leddet V. (2019). Acta Tropica Excito-repellent activity of β -caryophyllene oxide against *Aedes aegypti* and *Anopheles minimus*. Acta Trop..

[40] Sánchez-mendoza M.E., Cruz-antonio L., Cupido-sánchez M.G., García- G., Arrieta J. (2014). Gastroprotective activity of caryophyllene oxide: The role of nitric oxide, prostaglandins and sulfhydryls. J. Appl. Pharm. Sci..

[41] Dunkić V., Bezić N., Vuko E. (2011). Antiphytoviral Activity of Essential Oil from Endemic Species *Teucrium arduini*. Nat. Prod. Commun..

[42] Moreira R.R.D., dos Santos A.G., Carvalho F.A., Perego C.H., Crevelin E.J., Crotti A.E.M., Cogo J., Cardoso M.L.C., Nakamura C.V. (2019). Antileishmanial activity of *Melampodium divaricatum* and *Casearia sylvestris* essential oils on Leishmania amazonensis. Rev. Inst. Med. Trop. Sao Paulo.

[43] Benelli G., Govindarajan M., AlSalhi M.S., Devanesan S., Maggi F. (2018). High toxicity of camphene and γ-elemene from Wedelia prostrata essential oil against larvae of *Spodoptera litura* (Lepidoptera: Noctuidae). Environ. Sci. Pollut. Res..

[44] Govindarajan M., Rajeswary M., Senthilmurugan S., Vijayan P., Alharbi N.S., Kadaikunnan S., Khaled J.M., Benelli G. (2018). Curzerene, trans-β-elemenone, and γ-elemene as effective larvicides against *Anopheles subpictus*, *Aedes albopictus*, and *Culex tritaeniorhynchus*: Toxicity on non-target aquatic predators. Environ. Sci. Pollut. Res..

[45] Guo X., Shang X., Li B., Zhou X.Z., Wen H., Zhang J. (2017). Acaricidal activities of the essential oil from *Rhododendron nivale* Hook. f. and its main compund, δ-cadinene against Psoroptes cuniculi. Vet. Parasitol..

[46] Pérez-López A., Cirio A.T., Rivas-Galindo V.M., Aranda R.S., De Torres N.W. (2011). Activity against streptococcus pneumoniae of the essential oil and δ-cadinene isolated from schinus molle fruit. J. Essent. Oil Res..

[47] Ho C.L., Liao P.C., Wang E.I.C., Sua Y.C. (2011). Composition and antifungal activities of the leaf essential oil of Neolitsea parvigemma from Taiwan. Nat. Prod. Commun..

[48] Su Y.C., Hsu K.P., Wang E.I.C., Ho C.L. (2013). Composition and in vitro anticancer activities of the leaf essential oil of neolitsea variabillima from Taiwan. Nat. Prod. Commun..

[49] Ozkan G., Baydar H., Erbas S. (2010). The influence of harvest time on essential oil composition, phenolic constituents and antioxidant properties of Turkish oregano (*Origanum onites* L.). J. Sci. Food Agric..

[50] dos Santos J.F.S., Rocha J.E., Bezerra C.F., do Nascimento Silva M.K., de Matos Y.M.L.S., de Freitas T.S., dos Santos A.T.L., da Cruz R.P., Machado A.J.T., Rodrigues T.H.S. (2018). Chemical composition, antifungal activity and potential anti-virulence evaluation of the *Eugenia uniflora* essential oil against Candida spp.. Food Chem..

[51] Toledo A.G., de Souza J.G.D.L., da Silva J.P.B., Favreto W.A.J., da Costa W.F., Pinto F.G.D.S. (2020). Chemical composition, antimicrobial and antioxidant activity of the essential oil of leaves of Eugenia involucrata DC. Biosci. J..

[52] Mahboubi M. (2015). Chemical Composition, Antimicrobial andAntioxidant Activities of Eugenia caryophyllata Essential Oil. J. Essent. Oil Bear. Plants.

[53] Figueiredo P.L.B., Fernandes H.A., da Silva A.R.C., Alves N.S.F., Setzer W.N., da Silva J.K.R., Maia J.G.S. (2019). Variability in the Chemical Composition of Eugenia biflora Essential Oils from the Brazilian Amazon. Nat. Prod. Commun..

[54] Wootton-Beard P.C., Moran A., Ryan L. (2011). Stability of the total antioxidant capacity and total polyphenol content of 23 commercially available vegetable juices before and after in vitro digestion measured by FRAP, DPPH, ABTS and Folin-Ciocalteu methods. Food Res. Int..

[55] Santos D.N., De Souza L.L., De Oliveira C.A.F., Silva E.R., de Oliveira A.L. (2015). Arginase inhibition, antibacterial and antioxidant activities of Pitanga seed (*Eugenia uniflora* L.) extracts from sustainable technologies of high pressure extraction. Food Biosci..

[56] Xu K., Alves-Santos A.M., Dias T., Naves M.M.V. (2020). Grumixama (*Eugenia brasiliensis* Lam.) cultivated in the Cerrado has high content of bioactive compounds and great antioxidant potential. Ciência Rural.

[57] Celli G.B., Pereira-Netto A.B., Beta T. (2011). Comparative analysis of total phenolic content, antioxidant activity, and flavonoids profile of fruits from two varieties of Brazilian cherry (*Eugenia uniflora* L.) throughout the fruit developmental stages. Food Res. Int..

[58] Figueiredo P.L.B., Pinto L.C., da Costa J.S., da Silva A.R.C., Mourão R.H.V., Montenegro R.C., da Silva J.K.R., Maia J.G.S. (2019). Composition, antioxidant capacity and cytotoxic activity of *Eugenia uniflora* L. chemotype-oils from the Amazon. J. Ethnopharmacol..

[59] Oliveira D.C.S., Kaneko T.M., Young M.C.M., Murakami C., Cordeiro I., Moreno P.R.H. (2018). Chemical Composition, Antimicrobial and Antioxidant Activities of Eugenia Dysenterica DC Essential Oil. Emerg. Sci. J..

[60] Silvestri J.D.F., Paroul N., Czyewski E., Lerin L., Rotava I., Cansian R.L., Mossi A., Toniazzo G., de Oliveira D., Treichel H. (2010). Chemical composition and antioxidant and antibacterial activities of clove essential oil (*Eugenia caryophyllata* Thunb). Rev. Ceres.

[61] Carneiro N.S., Alves C.C.F., Alves J.M., Egea M.B., Martins C.H.G., Silva T.S., Bretanha L.C., Balleste M.P., Micke G.A., Silveira E.V. (2017). Chemical composition, antioxidant and antibacterial activities of essential oils from leaves and flowers of Eugenia klotzschiana Berg (Myrtaceae). An. Acad. Bras. Cienc..

[62] Gomes Vidal Sampaio M., Bezerra Dos Santos C.R., Cortez Sombra Vandesmet L., Souza Dos Santos B., Bianca Da Silva Santos I., dos Correia M.T.S., Lima de Berrêdo Martins A., Nascimento da Silva L.C., de Alencar Menezes I.R., Veja Gomez M.C. (2021). Chemical composition, antioxidant and antiprotozoal activity of Eugenia gracillima Kiaersk. leaves essential oil. Nat. Prod. Res..

[63] da Silva J., Andrade E., Barreto L., da Silva N., Ribeiro A., Montenegro R., Maia J. (2017). Chemical Composition of Four Essential Oils of Eugenia from the Brazilian Amazon and Their Cytotoxic and Antioxidant Activity. Medicines.

[64] Defaveri A.C.A., Sato A., Borré L.B., Aguiar D.L.M., Gil R.A.S.S., Arruda R.C.O., Riehl C.A.S. (2011). Eugenia neonitida sobral and *Eugenia rotundifolia* casar. (myrtaceae) essential oils: Composition, seasonality influence, antioxidant activity and leaf histochemistry. J. Braz. Chem. Soc..

[65] Shah B.B., Mehta A.A. (2018). In vitro evaluation of antioxidant activity of D-Limonene. Asian J. Pharm. Pharmacol..

[66] Hurtado F.B., Lima R.A., Teixeira L.F., Silva I.C., Bay M.B., Azevedo M.S., Facundo V.A. (2016). Antioxidant Activity and Characterization of the Essential Oil From the Roots of Piper Marginatum Jacq. Ciência Nat..

[67] Santana de Oliveira M., da Cruz J.N., Almeida da Costa W., Silva S.G., da Brito M.P., de Menezes S.A.F., de Jesus Chaves Neto A.M., de Aguiar Andrade E.H., de Carvalho Junior R.N. (2020). Chemical Composition, Antimicrobial Properties of Siparuna guianensis Essential Oil and a Molecular Docking and Dynamics Molecular Study of its Major Chemical Constituent. Molecules.

[68] Silva S.G., de Oliveira M.S., Cruz J.N., da Costa W.A., da Silva S.H.M., Barreto Maia A.A., de Sousa R.L., Carvalho Junior R.N., de Aguiar Andrade E.H. (2021). Supercritical CO2 extraction to obtain Lippia thymoides Mart. & Schauer (Verbenaceae) essential oil rich in thymol and evaluation of its antimicrobial activity. J. Supercrit. Fluids.

[69] van den Dool H., Kratz P.D. (1963). A generalization of the retention index system including linear temperature programmed gas—liquid partition chromatography. J. Chromatogr. A.

[70] Mondello L. (2011). FFNSC 2 Flavors and Fragrances of Natural and Synthetic Compounds 2 (Mass Spectral Database).

[71] Stein S., Mirokhin D., Tchekhovskoi D., Mallard G., Mikaia A., Zaikin V., Sparkmanm D. (2011). The NIST Mass Spectral Search Program for the Nist/Epa/Nih Mass Spectra Library.

[72] Miller N.J., Rice-Evans C., Davies M.J., Gopinathan V., Milner A. (1993). A Novel Method for Measuring Antioxidant Capacity and its Application to Monitoring the Antioxidant Status in Premature Neonates. Clin. Sci..

[73] Re R., Pellegrini N., Proteggente A., Pannala A., Yang M., Rice-Evans C. (1999). Antioxidant activity applying an improved ABTS radical cation decolorization assay. Free Radic. Biol. Med..

[74] BLOIS M.S. (1958). Antioxidant Determinations by the Use of a Stable Free Radical. Nature.

